# Analysis of the maternal inheritance hypothesis of the exochorium in eggs from hybrids of Chagas disease vectors

**DOI:** 10.1038/s41598-023-51125-w

**Published:** 2024-01-06

**Authors:** Paulo Sergio de Sousa, Jader de Oliveira, Amanda Ravazi, Yago Visinho dos Reis, Maria Tercília Vilela de Azeredo Oliveira, João Aristeu da Rosa, Cleber Galvão, Kaio Cesar Chaboli Alevi

**Affiliations:** 1 Institute of Biosciences (IBB), Sao Paulo State University (Unesp), Dr. Antonio Celso Wagner Zanin, 250, Botucatu, SP 18618-689 Brazil; 2 Laboratory of Entomology in Public Health, Department of Epidemiology, Faculty of Public Health (FSP), University of Sao Paulo (USP), Avenue Dr. Arnaldo, 715, Sao Paulo, SP 01246-904 Brazil; 3 Laboratory of Parasitology, School of Pharmaceutical Sciences (FCFAR), Sao Paulo State University (Unesp), Road Araraquara/Jau, Km 01, Araraquara, SP 14801-902 Brazil; 4 Laboratory of Cell Biology, Institute of Biosciences, Humanities and Exact Sciences (IBILCE), Sao Paulo State University (Unesp), Cristovao Colombo, 2265, Sao Jose do Rio Preto, SP 15054-000 Brazil; 5 National and International Reference Laboratory On Triatomine Taxonomy, Oswaldo Cruz Institute (IOC), Oswaldo Cruz Foundation (FIOCRUZ), Avenue Brasil, 4365, Rio de Janeiro, RJ 21040-360 Brazil

**Keywords:** Biological techniques, Zoology

## Abstract

Morphological studies applied to the taxonomy of the Triatominae cover various structures (head, wing, thorax, genitalia, and eggs). Exochorial structures of hybrid eggs were characterized and compared with the parents, demonstrating that hybrids presented characteristics identical to the exochorial pattern observed in the females of the crosses, which resulted in the hypothesis that the pattern of triatomine eggs is possibly a characteristic inherited from females. Thus, we characterized the exochorium of the eggs of several triatomine hybrids and compared them with the parents, to assess the pattern of segregation and test the hypothesis of maternal inheritance. Hybrids were obtained in at least one direction from all crosses. The analysis of the exochorium of the eggs of the hybrids showed different patterns of segregation: "exclusively paternal", "predominantly maternal", "predominantly paternal", "mutual", and "differential". Curiously, none of the hybrids evaluated presented characteristics that segregated exclusively from the female parental species. Thus, we demonstrate that the hypothesis of maternal inheritance of the exochorium pattern of eggs is not valid and we emphasize the importance of alternative/combined tools (such as integrative taxonomy) for the correct identification of these insect vectors (mainly in view of possible natural hybridization events due to climate and environmental changes).

## Introduction

Triatomines (Hemiptera, Triatominae) are hematophagous insects that act as vectors of the protozoan *Trypanosoma cruzi* (Chagas, 1909) (Kinetoplastida, Trypanosomatidae), the etiologic agent of Chagas disease (CD)^1,2^. This disease is considered neglected and affects about seven million people worldwide, causing approximately ten thousand deaths per year^1^. Although there are different forms of transmission, such as organ transplantation, blood transfusion, ingestion of contaminated food and laboratory accidents, the vector is considered the main route of transmission by the World Health Organization^1^.

Although the CD is curable if treatment with the antitrypanosomatides benznidazole and nifurtimox is initiated soon after infection (acute phase of the disease), the main way to minimize the incidence of new cases is based on the control of vector populations, as the acute phase is usually asymptomatic or causes nonspecific symptoms^1^. That way, studies related to triatomines are extremely important for public health, since they can generate subsidies to help vector control programs in the prophylaxis of CD^3^. Thus, since the first record in humans over 110 years ago^2^, several approaches have contributed to the biological, ecological, genetic, taxonomic, evolutionary and epidemiological knowledge of these vectors^4^.

Triatomines have the habit of defecating/urinating during or after a blood meal, thus releasing the parasite in the feces/urine if they are infected with *T. cruzi*^1^. There are 160 described species (157 living species and three fossil species), grouped into 18 genera and five tribes^5–9^, being all living species considered as potential vectors of CD. Currently, taxonomic studies of these vectors have been based on morphological, morphometric, genetic, cytogenetic, molecular analyses, and experimental crossings^5^. Morphological and morphometric studies applied to taxonomy cover various structures of triatomines, such as the head, wing, thorax and genitalia, as well as their eggs^10–20^

The eggs of these vectors have different structures, as lateral flattening, chorionic edge, opercular edge, neck, operculum, longitudinal bevel, collar, spermatic gutter, micropyles, aeropyles, sealing strip, hatching line, limiting lines, chorion, endochorion and exochorion, being many of these characteristics being used in taxonomic studies^18–27^. Recently, Sousa et al.^28^ grouped all dichotomous keys developed based on egg characteristics observed in light and scanning electron microscopy (SEM) and titled these keys EggKeys.

As mentioned above, among the different tools that can compound integrative taxonomy, carrying out experimental crosses and analysis of pre- and post-zygotic interspecific reproductive barriers are of great importance to assess the specific status of taxa (based on the biological species concept)^29,30^. Although most studies associated with experimental crossings are associated with taxonomy^3,21,31–49^, some researchers have studied the segregation pattern of phenotypic characteristics of triatomines in hybrids^11,22,31,38,41,42^. Only in 2014, morphological structures of hybrid eggs of *Triatoma lenti* Sherlock & Serafim, 1967 and *T. sherlocki* Papa, Jurberg, Carcavallo, Cerqueira & Barata, 2002 were characterized by SEM and compared with the parents^11^. These pioneering analyses into triatomines made it possible to observe that first-generation hybrids (F1) presented characteristics identical to the exochorion pattern observed in the females of the crosses, that is, the F1 hybrids from the cross between ♀ *T. lenti* and ♂ *T. sherlocki* showed an identical pattern to *T. lenti* and the cross between ♀ *T. sherlocki* and ♂ *T. lenti* showed an identical pattern to *T. sherlocki*^11^. According to these results, a hypothesis was raised that the pattern of triatomine eggs is possibly a characteristic inherited from females.

Based on the above, we characterized the exochorium of the eggs of several triatomine hybrids and compared them with the parents, to assess the pattern of segregation and test the hypothesis of maternal inheritance.

## Results

Hybrids were obtained in at least one direction from all crosses, i.e., between ♀ *Rhodnius robustus* Larrousse, 1927 and ♂ *R. prolixus* Stål, 1859, ♀ *R. prolixus* and ♂ *R. robustus*, ♀ *R. neivai* Lent, 1953 and ♂ *R. prolixus*, ♀ *R. prolixus* and ♂ *R. nasutus* Stål, 1859, ♀ *R. montenegrensis* Rosa et al., 2012 and ♂ *R. marabaensis* Souza et al., 2016, ♀ *R. marabaensis* and ♂ *R. montenegrensis*, ♀ *R. robustus* and ♂ *R. montenegrensis*, ♀ *R. montenegrensis* and ♂ *R. robustus*, ♀ *Psammolestes coreodes* Bergroth, 1911 and ♂ *P. tertius* Lent & Jurberg, 1965, ♀ *T. brasiliensis macromelasoma* Galvão, 1956 and ♂ *T. lenti*, ♀ *T. melanica* (Neiva & Lent, 1941) and ♂ *T. lenti*, as well as ♀ *T. lenti* and ♂ *T. juazeirensis* Costa & Felix, 2006.

**Figure 1:**
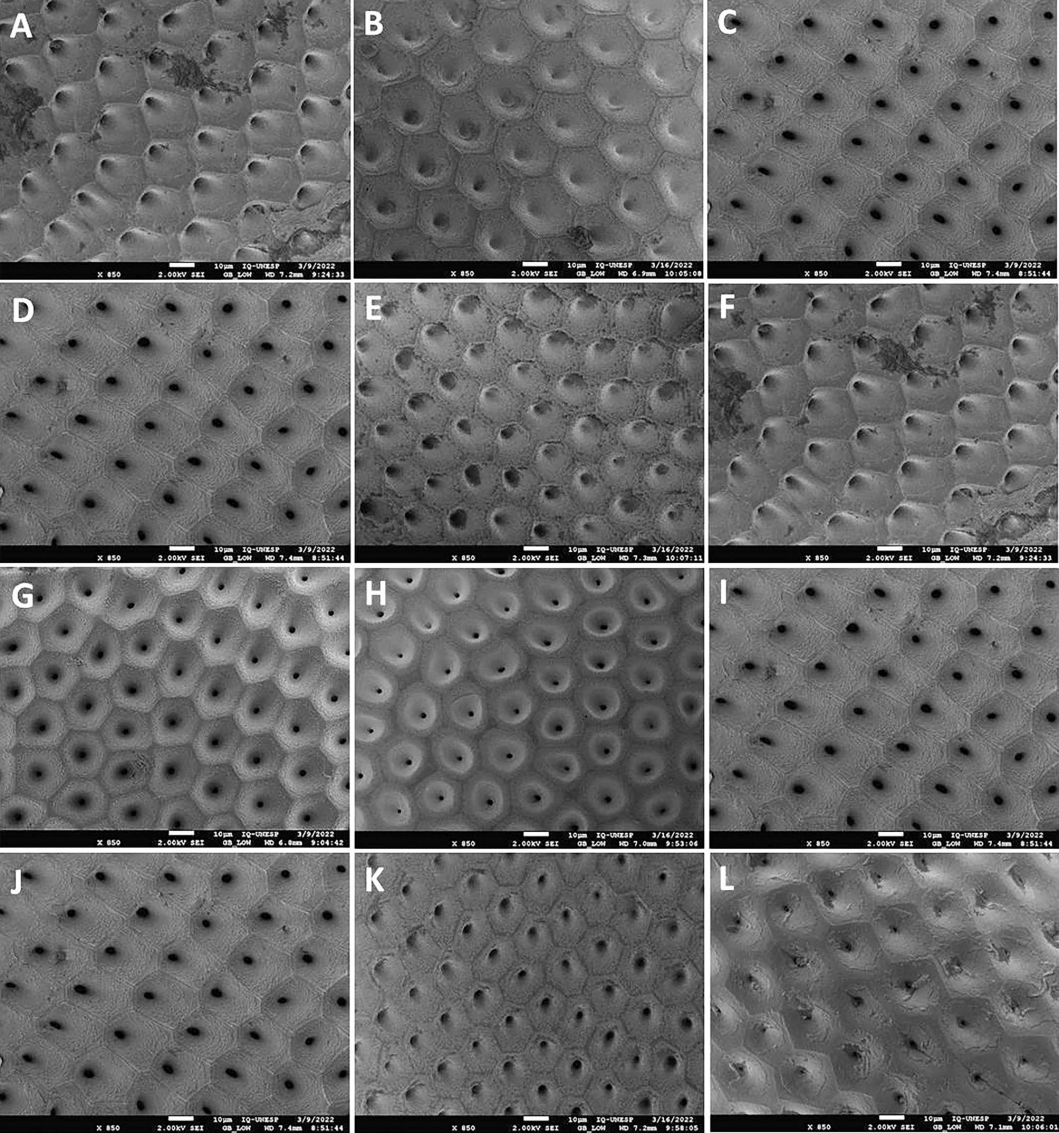
Characterization of the exochorium of the eggs of the parents and hybrids of *Rhodnius* spp. by SEM. (**A**,**F**) *R. robustus*, (**B**) Hybrid resulting from the cross between ♀ *R. robustus* × ♂ *R. prolixus*, (**C**,**D**,**I**,**J**) *R. prolixus*, (**E**) Hybrid resulting from the cross between ♀ *R. prolixus* × ♂ *R. robustus*, (**G**) *R. neivai*, (**H**) Hybrid resulting from the cross between ♀ *R. neivai* × ♂ *R. prolixus*, (**K**) Hybrid resulting from the cross between ♀ *R. prolixus* × ♂ *R. nasutus*, (**L**) *R. nasutus*.

**Figure 2:**
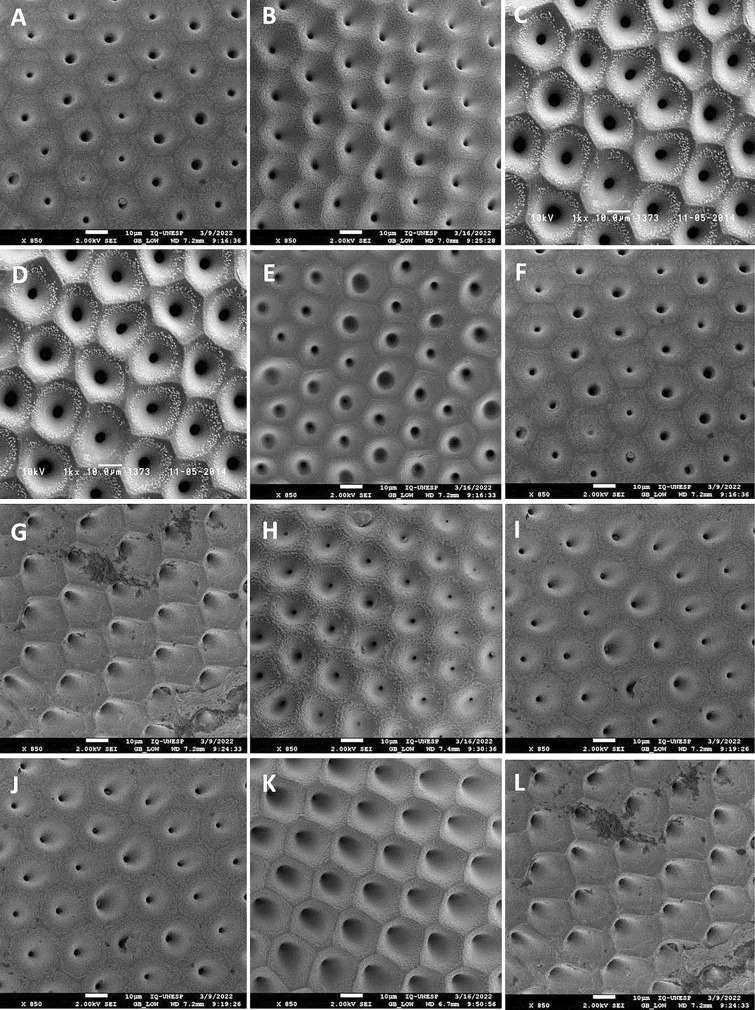
Characterization of the exochorium of the eggs of the parents and hybrids of *Rhodnius* spp. by SEM. (**A**,**F**,**I**,**J**) *R. montenegrensis*, (**B**) Hybrid resulting from the cross between ♀ *R. montenegrensis* × ♂ *R. marabaensis*, (**C**,**D**) *R. marabaensis*, (**E**) Hybrid resulting from the cross between ♀ *R. marabaensis* × ♂ *R. montenegrensis*, (**G**,**L**) *R. robustus*, (**H**) Hybrid resulting from the cross between ♀ *R. robustus* × ♂ *R. montenegrensis*, (**K**) Hybrid resulting from the cross between ♀ *R. montenegrensis* × ♂ *R. robustus*.

**Figure 3:**
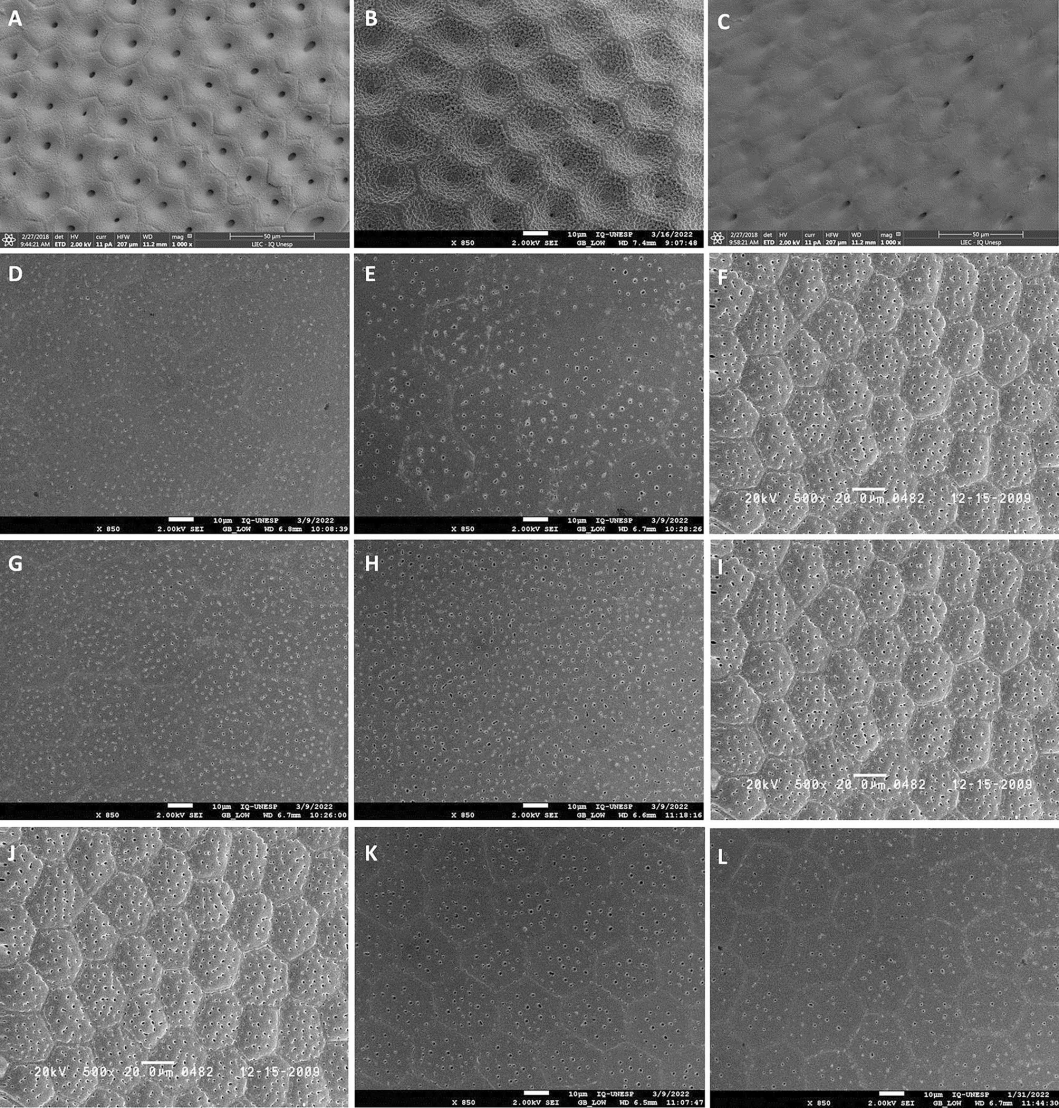
Characterization of the exochorium of the eggs of the parents and hybrids of *Psammolestes* spp. and *Triatoma* spp. by SEM. (**A**) *P. coreodes*, (**B**) Hybrid resulting from the cross between ♀ *P. coreodes* × ♂ *P. tertius*, (**C**) *P. tertius*, (**D**) *T. b. macromelasoma*, (**E**) Hybrid resulting from the cross between ♀ *T. b. macromelasoma* × ♂ *T. lenti*, (**F**,**I**,**J**) *T. lenti*, (**G**) *T. melanica*, (**H**) Hybrid resulting from the cross between ♀ *T. melanica* × ♂ *T. lenti*, (**K**) Hybrid resulting from the cross between ♀ *T. lenti* × ♂ *T. juazeirensis*, (**L**) *T. juazeirensis*.

The analysis of the exochorium of the eggs of the hybrids and the parents (Figs. 1, 2, 3 and Tables 1, 2, 3) showed different patterns of segregation:

**Table 1:** Morphological characteristics of the exochorion of the parents and segregation pattern in the hybrids of *Rhodnius* spp.

Characteristics	*R. robustus* (Fig. 1A)	*R. prolixus* (Fig. 1C)	Hybrid (Fig. 1B) (♀ *R. robustus* × ♂ *R. prolixus*)
Cells	Hexagonal	Pentagonal and hexagonal	Hexagonal
Limiting lines	Evident	Very evident (easy to see)	Very evident (easy to see)
Granulations	Irregular and less abundant, with greater concentration around the LL	Regular and abundant	Regular and abundant

Characteristics	*R. robustus* (Fig. 1F)	*R. prolixus* (Fig. 1D)	Hybrid (Fig. 1E) (♀ *R. prolixus* × ♂ *R. robustus*)
Cells	Hexagonal	Pentagonal and hexagonal	Hexagonal
Limiting lines	Evident	Very evident (easy to see)	Less evident
Granulations	Irregular and less abundant, with greater concentration around the LL	Regular and abundant	Irregular and less abundant, with greater concentration around the LL

Characteristics	*R. neivai* (Fig. 1G)	*R. prolixus* (Fig. 1I)	Hybrid (Fig. 1H) (♀ *R. neivai* × ♂ *R. prolixus*)
Cells	Pentagonal and hexagonal	Pentagonal and hexagonal	Pentagonal and hexagonal
Limiting lines	Very evident (easy to see)	Very evident (easy to see)	Less evident
Granulations	Regular and abundant	Regular and abundant	Regular and abundant, with greater concentration around the LL

Characteristics	*R. prolixus* (Fig. 1J)	*R. nasutus* (Fig. 1L)	Hybrid (Fig. 1K) (♀ *R. prolixus* × ♂ *R. nasutus*)
Cells	Pentagonal and hexagonal	Hexagonal and irregular	Hexagonal
Limiting lines	Very evident (easy to see)	Less evident	Evident
Granulations	Regular and abundant	Irregular and less abundant, frequently forming clumps	Regular and abundant

**Table 2:** Morphological characteristics of the exochorion of the parents and segregation pattern in the hybrids of *Rhodnius* spp.

Characteristics	*R. montenegrensis* (Fig. 2A)	*R. marabaensis* (Fig. 2C)	Hybrid (Fig. 2B) (♀ *R. montenegrensis* × ♂ *R. marabaensis*)
Cells	Pentagonal and hexagonal	Hexagonal and irregular	Hexagonal
Limiting lines	Evident	Evident	Less evident
Granulations	Regular and abundant	Regular and abundant, with greater concentration around the LL	Regular and abundant, with greater concentration around the LL

Characteristics	*R. montenegrensis* (Fig. 2F)	*R. marabaensis* (Fig. 2D)	Hybrid (Fig. 2E) (♀ *R. marabaensis* × ♂ *R. montenegrensis*)
Cells	Pentagonal and hexagonal	Hexagonal and irregular	Hexagonal and irregular
Limiting lines	Evident	Evident	Less evident
Granulations	Regular and abundant	Regular and abundant, with greater concentration around the LL	Irregular and less abundant

Characteristics	*R. robustus* (Fig. 2G)	*R. montenegrensis* (Fig. 2I)	Hybrid (Fig. 2H) (♀ *R. robustus* × ♂ *R. montenegrensis*)
Cells	Hexagonal	Pentagonal and hexagonal	Pentagonal and hexagonal
Limiting lines	Evident	Less evident	Less evident
Granulations	Irregular and less abundant, with greater concentration around the LL	Regular and abundant, with greater concentration around the LL	Regular and abundant, frequently forming clumps

Characteristics	*R. robustus* (Fig. 2L)	*R. montenegrensis* (Fig. 2J)	Hybrid (Fig. 2K) (♀ *R. montenegrensis* × ♂ *R. robustus*)
Cells	Hexagonal	Pentagonal and hexagonal	Hexagonal
Limiting lines	Evident	Less evident	Very evident (easy to see)
Granulations	Irregular and less abundant, with greater concentration around the LL	Regular and abundant, with greater concentration around the LL	Regular and abundant

**Table 3:** Morphological characteristics of the exochorion of the parents and segregation pattern in the hybrids of *Psammolestes* spp. and *Triatoma* spp.

Characteristics	*P. coreodes* (Fig. 3A)	*P. tertius* (Fig. 3C)	Hybrid (Fig. 3B) (♀ *P. coreodes* × ♂ *P. tertius*)
Cells	Pentagonal, hexagonal and irregular	Hexagonal and irregular	Pentagonal and hexagonal
Limiting lines	Less evident	Absent	Very evident (easy to see)
Granulations	Irregular and abundant, with less concentration around the LL	Regular and abundant	Regular and abundant

Characteristics	*T. b. macromelasoma* (Fig. 3D)	*T. lenti* (Fig. 3F)	Hybrid (Fig. 3E) (♀ *T. b. macromelasoma* × ♂ *T. lenti*)
Cells	Hexagonal and irregular	Pentagonal, hexagonal and irregular	Hexagonal
Limiting lines	Less evident	Evident	Less evident
Pores	Regular and abundant	Regular and abundant	Irregular and abundant

Characteristics	*T. melanica* (Fig. 3G)	*T. lenti* (Fig. 3I)	Hybrid (Fig. 3H) (♀ *T. melanica* × ♂ *T. lenti*)
Cells	Pentagonal, hexagonal and irregular	Pentagonal, hexagonal and irregular	Pentagonal and hexagonal
Limiting lines	Less evident	Evident	Absent
Pores	Regular and abundant	Regular and abundant	Regular and abundant

Characteristics	*T. juazeirensis* (Fig. 3L)	*T. lenti* (Fig. 3J)	Hybrid (Fig. 3K) (♀ *T. lenti* × ♂ *T. juazeirensis*)
Cells	Pentagonal, hexagonal and irregular	Pentagonal, hexagonal and irregular	Pentagonal, hexagonal and irregular
Limiting lines	Less evident	Evident	Less evident
Pores	Regular and less abundant	Regular and abundant	Regular and less abundant

i. "exclusively maternal" segregation: not observed in eggs of any of the hybrids.

ii. "exclusively paternal" segregation: hybrids resulting from crosses between ♀ *T. lenti* × ♂ *T. juazeirensis*.

iii. "predominantly maternal" segregation: hybrids resulting from crosses between ♀ *R. prolixus* × ♂ *R. robustus*.

iv. "predominantly paternal" segregation: hybrids resulting from crosses between ♀ *R. robustus* × ♂ *R. prolixus*, ♀ *R. montenegrensis* × ♂ *R. marabaensis*, and ♀ *R. robustus* × ♂ *R. montenegrensis*.

v. "mutual" segregation: hybrids resulting from crosses between ♀ *R. robustus* × ♂ *R. prolixus*, and ♀ *R. prolixus* × ♂ *R. nasutus*,

vi. "differential" segregation: hybrids resulting from crosses between ♀ *R. prolixus* × ♂ *R. robustus*, ♀ *R. neivai* × ♂ *R. prolixus*, ♀ *R. prolixus* × ♂ *R. nasutus*, ♀ *R. montenegrensis* × ♂ *R. marabaensis*, ♀ *R. marabaensis* × ♂ *R. montenegrensis*, ♀ *R. robustus* × ♂ *R. montenegrensis*, ♀ *R. montenegrensis* × ♂ *R. robustus*, ♀ *P. coreodes* × ♂ *P. tertius*, ♀ *T. b. macromelasoma* × ♂ *T. lenti*, and ♀ *T. melanica* × ♂ *T. lenti*.

## Discussion

Of the six segregation patterns evaluated, differential segregation was observed in most of the hybrid eggs (Tables 1, 2, 3). This phenomenon may result from the fact that hybrids are organisms resulting from the crossing of two different species^43^, that is, two distinct haploid genomes unite and, in general, can form a hybrid organism genotypically different from the parents^43^ (which may reflect in phenotypic characters not shared with the species that originated the hybrids).

Although Mendonça et al.^11^ observed exclusively maternal segregation for eggs of hybrids resulting from crosses between *T. lenti* and *T. sherlocki*, curiously, none of the hybrids evaluated in the present work presented characteristics of the morphology of the egg that segregated exclusively from the female species. This fact makes the hypothesis of maternal segregation of eggs unfeasible (being, therefore, only a peculiar characteristic of the hybrids analyzed by Mendonça et al.^11^).

Mendonça et al.^11^ evaluated the morphological segregation pattern of the median process of the pygophore in F1 hybrids and also observed segregation of characters that were not present in the parents (called intermediate characters by the authors). In addition, the authors evaluated the phenotype of the hybrids and observed different segregation patterns related to the size of the hemelytra (since *T. sherlocki* is brachyptera and unable to fly)^41^ and the color of the femoral rings. Pinotti et al.^42^ evaluated the segregation of morphological characters in hybrids of species of the *T. brasiliensis* subcomplex and observed that the hybrids resulting from the crosses between *T. b. brasiliensis* ♀ × *T. lenti* ♂, *T. juazeirensis* ♀ × *T. lenti* ♂, and *T. melanica* ♀ × *T. lenti* ♂ showed segregation of characteristics of both parental species. On the other hand, the hybrids between *T. lenti* ♀ × *T. juazeirensis* ♂, *T. b. macromelasoma* ♀ × *T. lenti* ♂, and *T. lenti* ♀ × *T. melanica* ♂ showed a specific pattern of *T. lenti*, *T. lenti* and *T. melanica*, respectively. In addition, a study using experimental crosses between *Mepraia spinolai* (Porter, 1934) and *M. gajardoi* Frias, Henry and Gonzalez, 1998 demonstrated that wingless males of *M. spinolai* produce both wingless and winged males^31^, thus demonstrating that the hypothesis of Frias and Átria^50^ that relate the genes linked to the wings with the Y sex chromosome is not valid—as the females of *Mepraia* spp. are always apterous or micropterous, the authors had suggested that the wing polymorphism present in males of *M. spinolai* would be related to a possible breakage of the Y sex chromosome (fragment Y1—specimens with wings and fragment Y2—specimens without wings).

Recently, Ravazi et al.^51^ evaluated the hybridization capacity of species from the Rhodniini tribe that live in sympatry and parapatry in the face of anthropogenic changes (environmental and climate changes). The authors observed that hybrids were produced in at least one direction of all crosses performed. This fact, when associated with the segregation patterns observed for the *Rhodnius* spp. and *Psammolestes* spp. (Tables 1, 2, 3), highlight the importance of other methodologies to confirm the specific status of species from the Rhodniini tribe, once climate and environmental changes may be influencing the dynamics of species distribution, which may lead to the formation of hybrids under natural conditions (thus making it difficult to use EggKeys^28^ for the correct identification of species).

This same issue may be happening with the species of the *T. brasiliensis* subcomplex, since entomoepidemiological studies and analyses of the distribution potential of this group of species in the face of climate change demonstrate that many taxa of this subcomplex have already been reported in sympatry^52–55^. These findings, when combined with the hybridization ability of most species in this subcomplex [except *T. petrocchiae* (Pinto & Barreto, 1925)]^3,36^ and with the pattern of egg segregation observed (Table 3), highlights the importance of alternative methodologies/keys in addition to the characterization of the egg exochorion^12,56^ for the taxonomy of the group.

Alevi et al.^5^ rescued the analyzes used in the description of the species of the Triatominae subfamily and observed that most taxa were described based on classical taxonomy (phenotypic analyses). However, the authors highlighted a trend towards the use of integrative taxonomy (which integrates morphological and morphometric studies with other approaches, such as molecular, ecological, behavioral, genetic, chromosomal, and reproductive, among others) in recent years. The genus *Rhodnius* Stål, 1859, in general, presents a very complex taxonomy, as cryptic speciation events and phenotypic plasticity have already been described in these triatomines^57,58^. Thus, the use of integrative taxonomy, as performed in the description of *R. montenegrensis*^10^, *R. barretti* Abad-Franch et al.^57^ and *R. marabaensis*^13^, allows greater reliability of the specific status of the species of this genus.

Likewise, several events of description, redescription, and synonymization have already been carried out in the *T. brasiliensis* subcomplex^12,59–63^. *Triatoma bahiensis* Sherlock & Serafim, 1967, for example, was described in 1967^64^, synonymized with *T. lenti* in 1979^50^ and only in 2016 was it revalidated, based on integrative taxonomy^12^. Several comparatives analyses between *T. bahiensis* and *T. lenti* were carried out (including the study of the exochorion of eggs in SEM). Although morphological and genetic differences were observed, experimental crosses were essential to confirm the specific status of *T. bahiensis*.

Thus, we demonstrate that the hypothesis of maternal inheritance of the exochorium pattern of eggs is not valid, and, above all, we emphasize the importance of alternative/combined tools (such as integrative taxonomy) for the correct identification of these insect vectors (mainly in view of possible natural hybridization events due to climate and environmental changes).

## Methods

### Experimental crosses

In order to obtain the eggs of the hybrids, interspecific crosses were performed between *R. robustus* and *R. prolixus* (both directions), *R. neivai* and *R. prolixus* (♀ *R. neivai* × ♂ *R. prolixus*), *R. prolixus* and *R. nasutus* (♀ *R. prolixus* × ♂ *R. nasutus*), *R. montenegrensis* and *R. marabaensis* (both directions), *R. robustus* and *R. montenegrensis* (both directions), *P. coreodes* and *P. tertius* (♀ *P. coreodes* × ♂ *P. tertius*), *T. b. macromelasoma* and *T. lenti* (♀ *T. b. macromelasoma* × ♂ *T. lenti*), *T. melanica* and *T. lenti* (♀ *T. melanica* × ♂ *T. lenti*), as well as *T. juazeirensis* and *T. lenti* (♀ *T. lenti* × ♂ *T. juazeirensis*). The species used were provided by the Triatominae Insectarium of the School of Pharmaceutical Sciences (FCFAR/UNESP), Araraquara, São Paulo, Brazil, where the crossings were also carried out. To ensure the virginity of the tested insects, fifth instar nymphs were separated and sexed^11^. After reaching the adult stage, the crosses were initiated and lasted 4 months^40^. Insect feeding were performed weekly during this period. The insects were kept at room temperature (average of 24 °C) and relative humidity of 63%^65^. After the hybrids reached the adult phase, intercrosses (♀ hybrid × ♂ hybrid) were performed to obtain the hybrid eggs. In addition, intraspecific crosses were performed to obtain eggs from the parents.

### Study of the exochorium of eggs in SEM

For the SEM analyses, ten eggs of each of the 12 parental species used in the crosses and of the 12 hybrids resulting from the interspecific crosses were prepared and analyzed in SEM, according to Mendonça et al.^11^: cleaned in an ultrasound machine, dehydrated in alcoholic series, dried in an oven at 45° for 20 min and then fixed in small aluminum cylinders “stubs” with colorless enamel. Subsequently, they were metalized by “sputtering” for 2 min with a power of 10 mA in a “Sputter” SCD 050 device and, finally, they were analyzed and photo-documented in SEM Topcon SM-300 (total magnification of 850 ×).

### Analyzed structures and classification of the segregation pattern

Among the different structures that make up the exochorium^25,26^, we analyzed the pattern of segregation of exochorial cells, limiting lines and granulation (for *Rhodnius*) or pores (for *Triatoma* Laporte, 1832).

The classification of the segregation pattern of the phenotypic characteristics of the exochorion was carried out as follows:

i. "exclusively maternal" segregation, when all the characteristics of the eggs of the hybrids are the same as those of the female species used in the crosses;
ii. "exclusively paternal" segregation, when all the characteristics of the eggs of the hybrids are the same as the male species used in the crosses;
iii. "predominantly maternal" segregation, when most of the characteristics of the eggs of the hybrids (two or more) are the same as the female species used in the crosses;
iv. "predominantly paternal" segregation, when most of the characteristics of the eggs of the hybrids (two or more) are the same as the male species used in the crosses;
v. "mutual" segregation, when at least one characteristic of the eggs of the hybrids is inherited from each of the parents;
vi. "differential" segregation, when at least one of the characteristics of the eggs of the hybrids is different from those observed in the parents.

## Data Availability

All relevant data are within the manuscript.
